# Safety and efficacy of the novel Alpha stent for the treatment of intracranial wide-necked aneurysm

**DOI:** 10.1038/s41598-024-59363-2

**Published:** 2024-04-16

**Authors:** Junhyung Kim, Jung-Jae Kim, Seung Won Kim, Jinyoung Choi, Hanki Kim, Jinwoo Kim, Joonho Chung

**Affiliations:** 1 Department of Neurosurgery, Gangnam Severance Hospital, Yonsei University College of Medicine, 20 Eonju-Ro 63-Gil, Gangnam-Gu, Seoul, 06229 Republic of Korea; 2 Department of Neurosurgery, Severance Hospital, Yonsei University College of Medicine, Seoul, Republic of Korea; 3 Department of Medical Sciences, Graduate School of Medicine, Korea University, Seoul, Republic of Korea; 4 Department of Bionanosystem Engineering, Graduate School, Jeonbuk National University, Jeonju, Republic of Korea; 5 Severance Hospital, Yonsei University Healthcare System, Seoul, Republic of Korea

**Keywords:** Phase IV trials, Stroke

## Abstract

The Alpha stent is an intracranial closed-cell stent with a unique mesh design to enhance wall apposition. It recently underwent structural modifications to facilitate easier stent deployment. This study aimed to evaluate the safety and efficacy of stent-assisted coil embolization for unruptured intracranial aneurysms using the Alpha stent. Between January 2021 and November 2021, 35 adult patients with 35 unruptured intracranial aneurysms in the distal internal carotid artery were prospectively enrolled. For efficacy outcomes, magnetic resonance angiography at the 6-month follow-up was evaluated using the Raymond-Roy occlusion classification (RROC). The safety outcome evaluated the occurrence of symptomatic procedure-related neurological complications up to 6 months postoperatively. Technical success was achieved in 34/35 (97.1%). Six months postoperatively, aneurysm occlusion showed RROC I in 32/35 (91.4%) and RROC II in 3/35 (8.6%) patients. Procedure-related neurologic complications occurred in one patient (2.9%) who experienced hemiparesis due to acute lacunar infarction, which resulted in a 6-month mRS score of 1. The Alpha stent demonstrated excellent efficacy and safety outcomes in stent-assisted coil embolization of unruptured distal ICA aneurysms. The recent structural modifications allowed for easier stent delivery and deployment.

**Clinical trial registration number**: KCT0005841; registration date: 28/01/2021.

## Introduction

Endovascular treatment is currently the dominant treatment modality for both ruptured and unruptured intracranial aneurysms (IAs)^1,2^. Wide-necked aneurysms, which are often unfeasible for simple coiling, can be effectively treated by stent-assisted coiling (SAC)^3–5^. Several stents are available for use in the treatment of IAs, and it is crucial to understand their physical properties, such as wall apposition, conformability, and foreshortening, which may affect the technical nuances of stent deployment^6^. For example, open-cell stents generally demonstrate better wall apposition than closed-cell stents, but they cannot be recaptured. Good wall apposition and the ability to be recaptured of a stent is especially important in cases of SAC involving distal internal carotid artery (ICA) due to the curvatures exhibited by the anterior genu portion or in the communicating segment^7^.

The Alpha stent (CGBio Co., Ltd., Seoul, Republic of Korea) is a recently developed, laser-cut, modified closed-cell stent that aims to capture the merits of both an open-cell stent and a closed-cell stent: good wall apposition and the ability to be recaptured. Its safety and 6-month follow-up efficacy results have been reported previously^7^. The authors also addressed the difficulties with stent deployment at intended locations. Subsequently, the Alpha stent underwent structural modifications aimed at enhancing safety and manipulability of stent deployment, including (1) reducing the length of the distal tip of a pusher guide wire by 25%, (2) increasing the bending force of the pusher guide wire by 50% for improved pushability, and (3) modifying the austenite finish temperature of the stent to prevent abrupt radial expansion during unsheathing. Here, we present the clinical and 6-month radiological follow-up results of 35 patients treated for unruptured IAs in distal ICA via SAC using the modified Alpha stent.

## Methods

This prospective, single-center, open-label, single-arm study evaluated the safety and effectiveness of stent-assisted coil embolization of wide-necked aneurysms, approved by Severance Hospital Institutional Review Board (1-2020-0077). The study was registered with the Clinical Research Information Service (No. KCT0005841, 28/01/2021). Informed consent was obtained from each participant prior to enrollment. The study was performed under the guidelines outlined by the Declaration of Helsinki and followed Standard Protocol Items: Recommendations for Interventional Trials checklist.

### Inclusion and exclusion criteria

The inclusion criteria were as follows: (1) age between 19 and 80 years and willingness to participate, (2) harboring unruptured saccular IAs in the distal ICA, and (3) having wide-necked aneurysms (neck ≥ 4 mm or dome-to-neck ratio < 2). Only distal ICA aneurysms were included because the Alpha Jr., was indicated for narrower vessels, and it was not a part of this study. The exclusion criteria entailed the following: (1) ruptured IAs or aneurysms associated with other cerebrovascular diseases such as moyamoya disease or arteriovenous malformation; (2) intracranial tumors or head and neck tumors receiving radiation therapy; (3) cardiac diseases such as atrial fibrillation associated with an increased risk of thromboembolic complications; and (4) contraindication for the use of anticoagulant or antiplatelet medication.

### Alpha stent description

**Figure 1:**
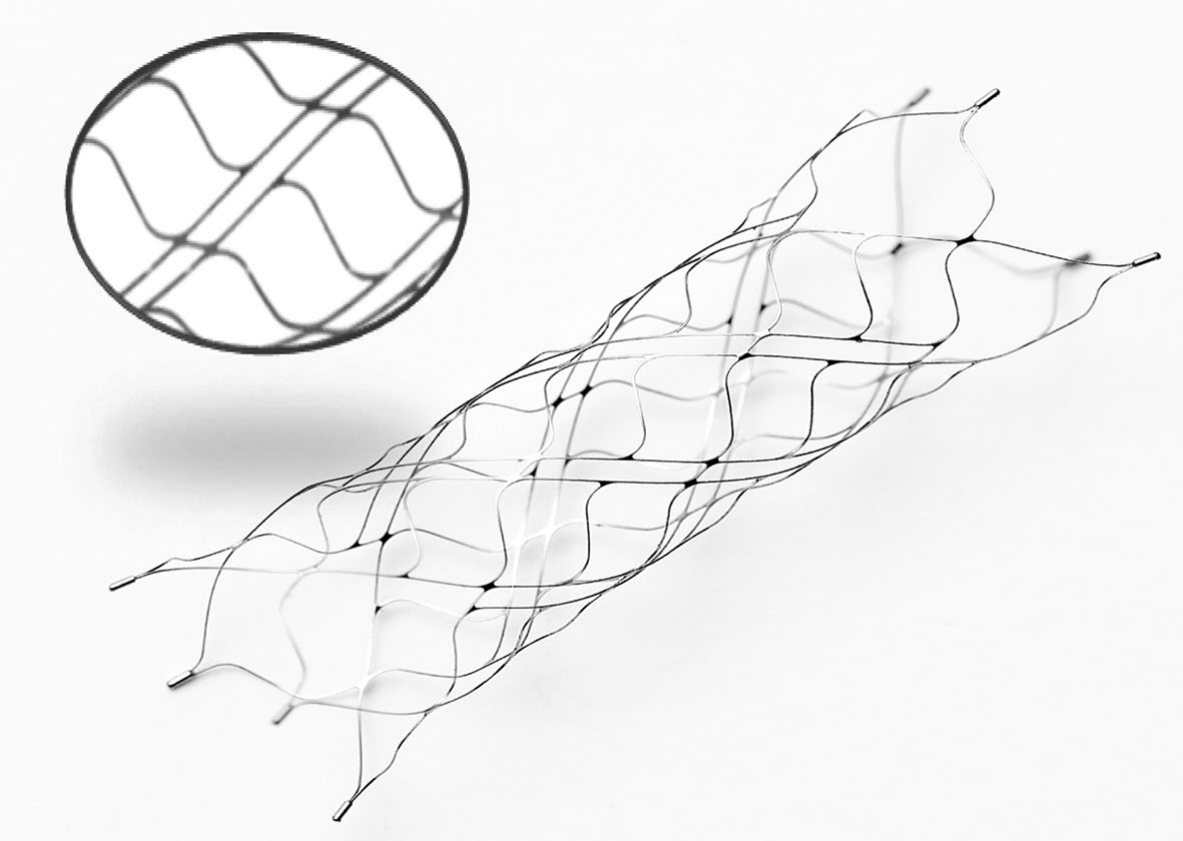
The stent demonstrates a closed-cell design with alternating columns of wide and short cells, and narrow and elongated cells. The columns of the elongated and narrow cells confer additional flexibility and enhanced wall apposition in curved vascular lesions.

The Alpha stent is a self-expandable, laser-cut intracranial stent made from a nitinol tube. It is characterized by a hybrid cell design composed of alternating columns of wide closed cells and narrow elongated cells (Fig. 1). Elongated cells exhibited longitudinal flexibility. This unique cell design is intended to capture the advantage of a closed-cell stent to provide the ability to be recaptured as well as that of an open-cell stent with superior wall apposition. Four radiopaque platinum markers are present at each end. The stent is 5.0 mm in diameter and 17 or 22 mm in length. It is indicated for use in a parent vessel ranging between 3.5 mm and 4.5 mm, and is delivered in a 0.021-inch microcatheter. Alpha Jr., is a low-profile stent with 3.5 mm in diameter, indicated for use in a parent vessel ranging between 2.0 mm and 3.5 mm, and is delivered in a 0.0165-inch microcatheter.

### Procedure description

Patients were administered oral aspirin (100 mg/day) and clopidogrel (75 mg/day) for at least 5 to 7 days prior to the scheduled endovascular procedure. All procedures were performed under general anesthesia, and vascular access was achieved via the femoral artery.

A stent with a 5-mm diameter was delivered via a 0.021-inch microcatheter (Headway^®^ 21; Microvention, Tustin, CA, USA or Prowler plus^®^; Codman Neurovascular, Raynham, MA, USA) and was deployed using a standard procedure. At the end of each procedure, a control angiogram was performed to evaluate immediate postprocedural aneurysm occlusion. The coiling strategies included the jailing and through-the-strut techniques. During the procedure, each patient received 50 IU/kg of intravenous heparin after puncture of the femoral artery, and an additional 1000 IU was administered every hour. The dual antiplatelet regimen was maintained for three months post-procedure, followed by aspirin monotherapy until the end of this study. At the end of each procedure, angiography was performed to evaluate immediate postprocedural aneurysm occlusion using the Raymond-Roy occlusion classification (RROC), in which RROC I was defined as complete occlusion, RROC II as a neck remnant, and RROC III as a sac remnant^8^.

### Clinical and radiologic follow-up

Patients (35 patients) visited the outpatient clinic at 1 month ± 2 weeks and 6 ± 1 months post-procedure for clinical assessment. At the 6 month follow-up, non-contrast brain MR angiography (MRA) was performed to determine the rate of aneurysm occlusion assessed using RROC. The RROC is the standard for evaluating coiled aneurysms (Class I: complete obliteration; Class II: residual neck; Class III: residual aneurysm)^8^. Two neurointerventionists (JC and JK) assessed the final MRA for the outcome evaluation and consensus was sought when the two neurointerventionists assessed differently.

### Primary and secondary outcomes

The primary efficacy outcome was the rate of successful aneurysm occlusion 6 months post- procedure, defined as RROC 1 (complete obliteration; total occlusion with no residual lumen filling) or 2 (residual neck; 95% occlusion but minimal residual filling with coils at the aneurysm neck) evaluated by MRA. Secondary safety endpoints were (1) the incidence of cerebrovascular complications within 6 months following the procedure, including symptomatic ischemic stroke (transient or permanent) or intracranial hemorrhage, rupture of the target aneurysm, and occlusion of the parent artery or adjacent branches; (2) incidence of device-related serious adverse events that result in death, permanent disability, extension of the hospitalization period longer than 3 days or readmission, (3) target aneurysm retreatment rate, and (4) technical success defined as successful stent deployment in the parent artery covering the aneurysmal neck.

### Statistical analysis

Data were presented as medians and interquartile ranges, or percentages with 95% confidence intervals for continuous and categorical variables, respectively.

## Results

### Patient and aneurysm characteristics

**Figure 2:**
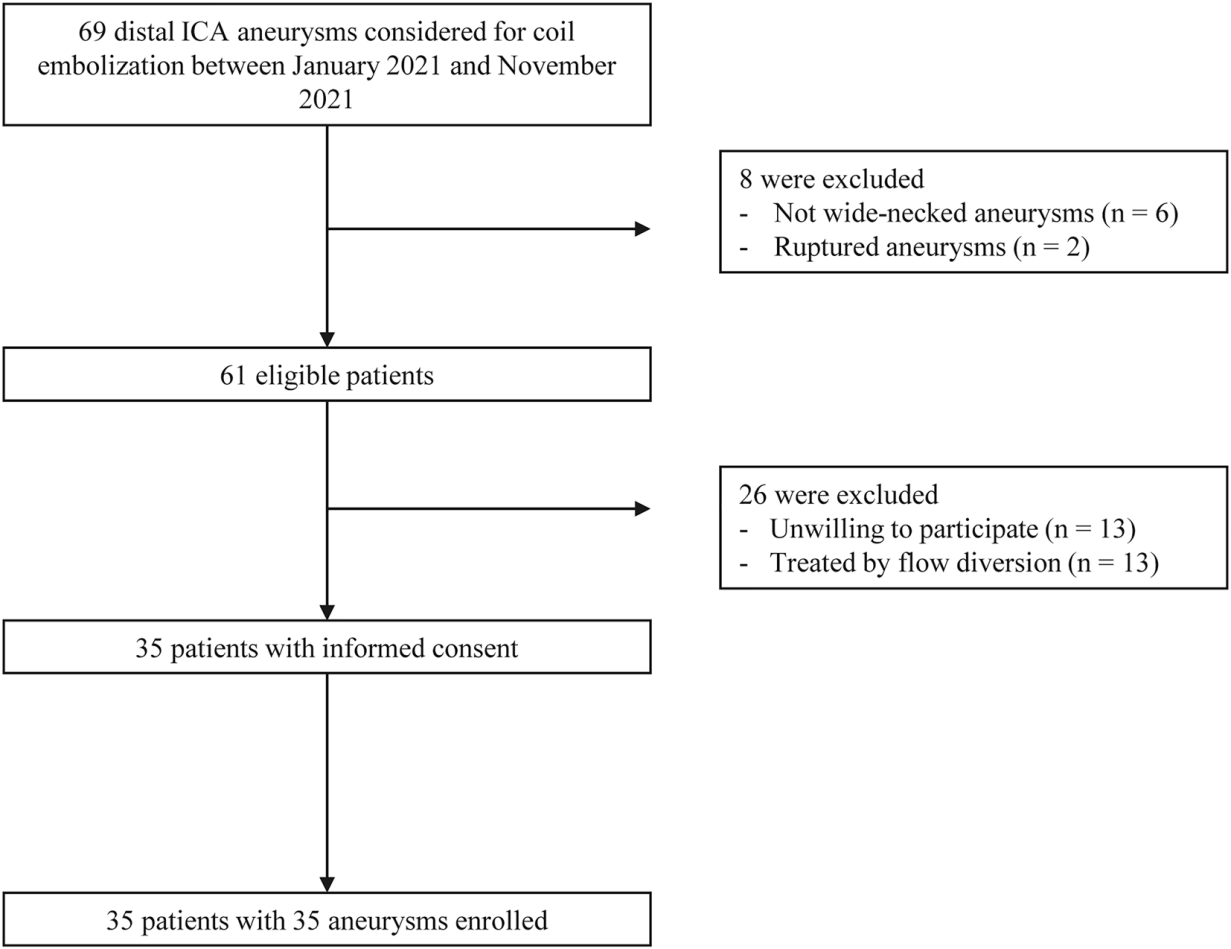
Flow chart for patient enrollment.

Thirty-five patients with 35 unruptured IAs were prospectively enrolled between January 2021 and November 2021 (Fig. 2). The median value for the maximum diameter was 5.22 mm and mean dome-to-neck ratio was 1.17. Most aneurysms were located in the ICA-paraclinoid segment (n = 29; 82.9%). One patient was treated for an ICA-cavernous segment aneurysm, because the patient had a family history of aneurysmal subarachnoid hemorrhage. Thirty-four aneurysms (97.1%) were treated using either the jailing technique (n = 31, 88.6%) or through-the-strut technique (n = 3, 8.6%). The baseline and procedural characteristics are summarized in Table 1.

**Table 1:** Patient, aneurysm, and procedural characteristics.

Variables	Values (n = 35)
Patients	
Age, median (interquartile range)	60 (51–64)
Female, n (%)	31 (88.6)
Aneurysms	
Location, n (%)	
ICA, cavernous segment	1 (2.9)
ICA-ophthalmic artery	1 (2.9)
ICA, paraclinoid segment	29 (82.9)
ICA-posterior communicating artery	4 (11.4)
Aneurysm size, mm (interquartile range)	
Maximum	5.22 (4.18–6.63)
Dome	4.16 (3.25–4.83)
Neck	3.41 (2.94–3.97)
Dome-to-neck ratio	1.17 (1.04–1.37)
Procedures	
Stent placement technique, n (%)	
Jailing	31 (88.6)
Through-the-strut	3 (8.6)
Bailout	1 (2.9)
Stent size, n (%)	
5 × 17 mm	26 (74.3)
5 × 22 mm	9 (25.7)

### Procedural events

Technical success was achieved in 34 of the 35 cases (97.1%). Stent migration to the proximal portion of the ICA occurred in one case, as the pusher guide wire dragged the fully deployed stent along with it during its retrieval. Incomplete occlusion of the aneurysm was achieved without using another stent. The aneurysm was treated by coiling without a stent, resulting in immediate postprocedural occlusion of the RROC II.

A device-related problem, not regarded as a technical failure occurred in one case. The proximal end of the stent was not fully deployed during coil embolization of the ICA-paraclinoid aneurysm, although no additional procedure was necessary.

### Radiologic and clinical outcomes

Aneurysm occlusion assessed immediately after the procedure showed RROC I in 24 aneurysms (68.6%), RROC II in eight aneurysms (22.9%), and RROC III in three aneurysms (8.5%). Radiologic follow-up at 6 months was achieved in all 35 patients and revealed RROC I in 34 aneurysms (97.1%) and RROC II in one aneurysm (2.9%).

All 35 patients were clinically followed up. Procedure-related complications occurred in one patient after an uneventful stent-assisted coil embolization of the right ICA-paraclinoid aneurysm. The patient complained of mild left hemiparesis 24 h postprocedurally, and diffusion-weighted imaging demonstrated acute lacunar infarction in the right corona radiata, resulting in an mRS score of 1 at the 6-month follow-up visit. No hemorrhagic procedure-related complications, morbidity, or mortality were observed. The radiological and clinical outcomes are presented in Table 2.

**Table 2:** Radiologic and clinical outcomes.

Radiologic outcomes	Values
Immediate postprocedural occlusion, n (%)	
RROC I	23 (65.7)
RROC II	9 (25.7)
RROC III	3 (8.6)
6-month follow-up occlusion, n (%)	
RROC I	32 (91.4)
RROC II	3 (8.6)
RROC III	0
Procedure-related complications, n (%)	
Ischemic	
Acute lacunar infarct	1 (2.9)*
Hemorrhagic	None

*RROC* Raymond-Roy occlusion classification.

*The patient expressed subjected left leg weakness (mRS score 1).

## Discussion

This single-center prospective study of coil embolization with the novel Alpha stent for the treatment of wide-necked ICA aneurysms revealed a technical success rate of 97.1% and a complete occlusion rate at 6 months of 91.4%, with a 2.9% procedure-related complication rate and no morbidity or mortality.

SAC for wide-necked aneurysms using various stents has shown excellent occlusion rates. For the closed-cell Enterprise stent, Jia et al. reported a complete occlusion rate and a near-complete occlusion rate of 80.8% and 13.8%, respectively, at a mean of 8 months^9^. Another study using Enterprise stent revealed an approximately 95% complete or near-complete occlusion rate at a mean of 11.9 months^10^. Complete or near-complete occlusion rates for the Neuroform Atlas (Stryker Neurovascular, Fremont, California, USA) were reported between 91.5 and 98.9% at a 12-month follow-up^11–13^. For the LVIS stent, Iosif et al.^14^ reported a complete or near-complete occlusion rate of 98.5% at 18 months, and Shankar et al.^15^ presented 88% of complete or near-complete occlusion without a necessity for retreatment at a median follow-up of 1 year. Song et al. reported the outcomes of the first clinical study on the safety and efficacy of the Alpha stent in 50 patients with 54 unruptured aneurysms^7^. The authors demonstrated that complete or near-complete aneurysm occlusion at 6-month was achieved in 52/54 aneurysms (96.2%), while two aneurysms (3.7%) required retreatment. The current study revealed the high efficacy of the Alpha stent in SAC of unruptured IAs with a 91.4% complete occlusion rate at 6 months, although direct comparisons with aforementioned studies using other types of stents may not be reasonable because the current study was limited to aneurysms in distal ICA.

According to recent studies, procedure-related neurological complications associated with SAC of unruptured or remotely ruptured aneurysms occur in 2.2–15.2%^16–19^. The first clinical study using the Alpha stent reported a 10% periprocedural ischemic complication rate, which also included transient ischemic attacks without brain lesions, without mortality or permanent morbidity at the 6-month follow-up. In the current study, one patient (2.9%) experienced symptomatic lacunar infarction that resulted in a 6-month mRS score of 1. Our findings suggest that the safety profile of SAC with the Alpha stent is comparable to that observed in prior studies with other types of stents. A long-term follow-up study including ruptured aneurysms may provide more detailed information regarding its safety profile.

The technical success rate was 97.1% (n = 34/35). Stent migration during pusher guide wire retrieval, as described in a previous study, also occurred in one patient in this study. Song et al. indicated that the abrupt diametric changes at the junction between the pusher wire and the distal tip might have served as a latch responsible for stent migration during pusher wire retrieval^7^. The mechanism for such a phenomenon differs from that of delayed stent migration, which largely resulted from a discrepancy in the proximal and distal vessel diameters^20,21^. Further investigation and appropriate structural modifications are necessary to prevent the proximal migration of the Alpha stent upon the removal of the pusher wire.

**Figure 3:**
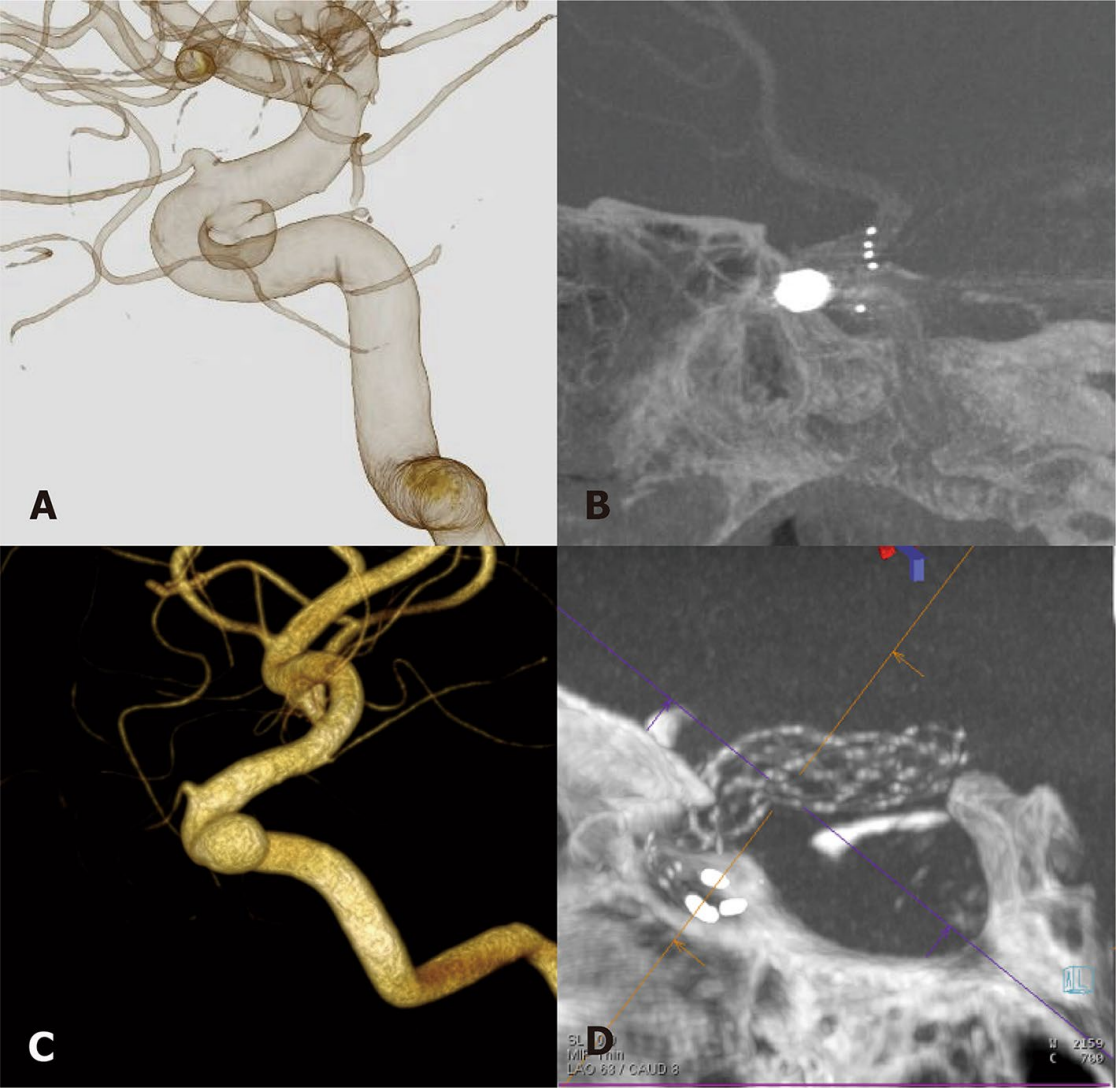
Two representative cases demonstrate successful deployments of the Alpha stents for treating ICA paraclinoid aneurysms. (**A**) and (**C**) 3-dimensional rotational angiograms of right ICA paraclinoid aneurysms. (**B**) and (**D**) Flat-panel CT images depict good wall apposition of the stents in the anterior genu of the ICA.

The main advantage of the Alpha stent stems from its hybrid cell design. In our experience, stents can be successfully recaptured without much difficulty. At the same time, they demonstrated excellent wall apposition and no kinking, especially when deployed in the paraclinoid ICA involving the curve of the anterior genu (Fig. 3). These features make the Alpha stent particularly useful in tortuous parent arteries, as a closed-cell stent system reportedly yields insufficient wall apposition in the tortuous parent vessels^22,23^. Our initial experience with the Alpha stent is confined to ICA aneurysms. However, analysis of its application in other locations such as proximal anterior cerebral artery/middle cerebral artery and the vertebrobasilar artery, as well as the utilzation of the Alpha Jr. stent in smaller arteries, warrants further investigation.

A few problems associated with the deployment of the Alpha stent were addressed by Song et al.^7^ Stent deployment at the intended site was not accomplished at the first attempt in five cases because unintended microcatheter displacement occurred during the deployment. In three patients, the stents did not fully open. Modifications to the stent properties and delivery system were subsequently performed to decrease the pushing force associated with stent deployment and thus facilitate smoother stent deployment by increasing the bending force of the pusher wire and adjusting the abrupt radial expansion upon stent unsheathing. In our study, with the structural revisions that enhanced the ease of stent delivery, no unintended microcatheter displacement was observed during stent unsheathing and only one case of inadvertent partial opening of the proximal segment of the stent.

**Figure 4:**
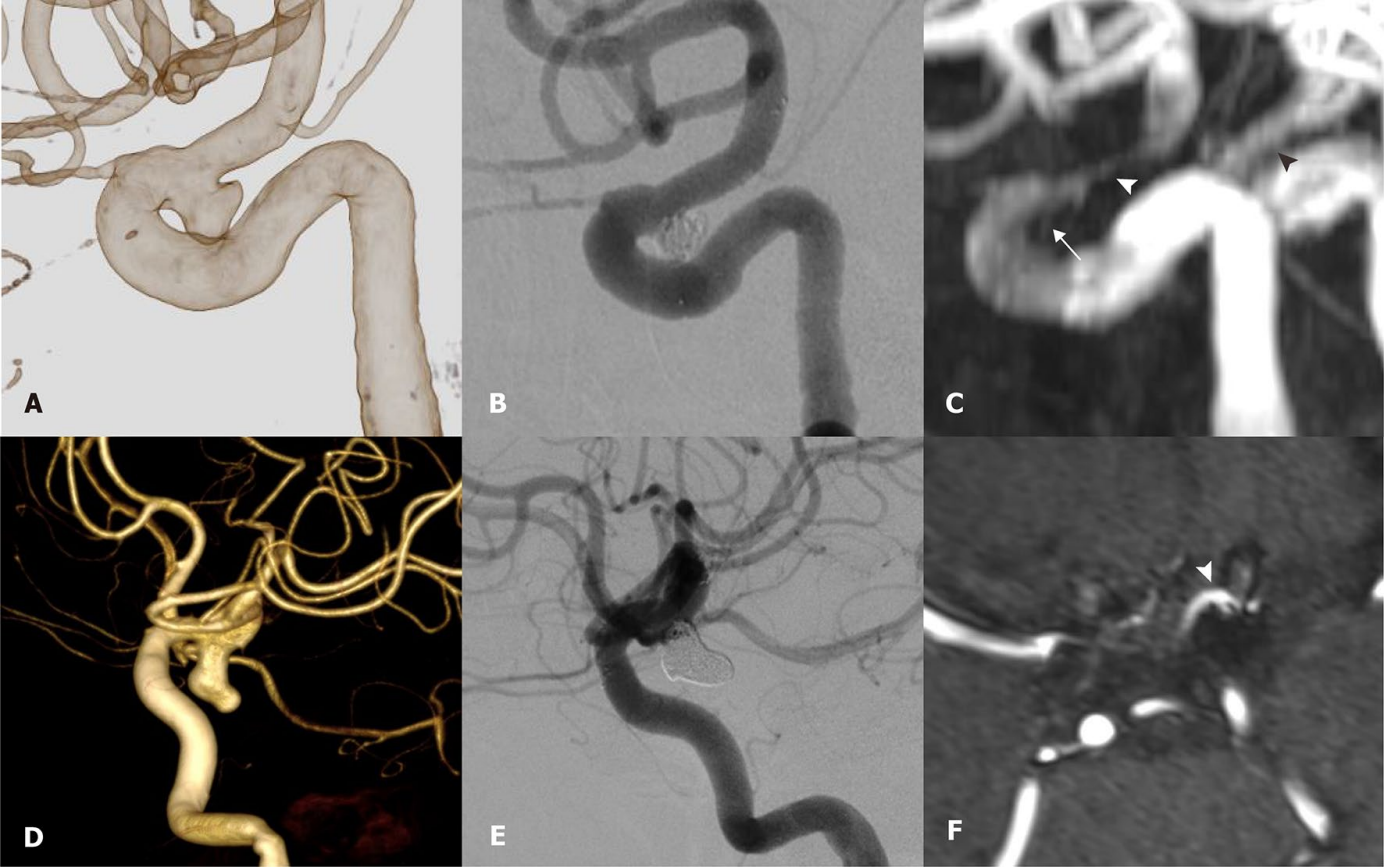
Visibility of the stent and adjacent vessels on TOF-MRA. (**A**) 3-dimensional rotational angiogram of left ICA shows a saccular wide-necked aneurysm at the paraclinoid segment. (**B**) Control angiogram after stent-assisted coil embolization reveals small aneurysmal neck remnant. (**C**) TOF-MRA taken 6 months after the procedure shows a residual neck remnant (white arrow) and a significant reduction of the in-stent signal (white arrowhead). A black arrowhead indicates less in-stent signal reduction in the contralateral ICA, where Neuroform Atlas was previously deployed. (**D**) 3-dimentional rotational angiogram of left ICA shows a 8-mm aneurysm at the ICA-posterior communicating artery junction. (**E**) Control angiogram after stent-assisted coil embolization reveals the preservation of posterior communicating artery flow. (**F**) TOF-MRA taken 6 months after the procedure shows in-stent signal reduction but a patent posterior communicating artery (white arrowhead).

As noted by Song et al.^7^, we observed metal artifacts that led to the reduction of the in-stent signal of the Alpha stent in time-of-flight MRA (TOF-MRA). In one patient for whom the Alpha stent and the Neuroform Atlas were deployed in each ICA, TOF-MRA showed significantly higher in-stent signal reduction for the Alpha stent (Fig. 4). Choi et al. demonstrated that open-cell stents [Neuroform and Wingspan (Stryker Neurovascular, Fremont, California, USA)] were less susceptible to MR artifacts than closed-cell stents [Solitaire (Medtronic, Irvine, CA, USA) and Enterprise (Codman Neurovascular, Raynham, MA, USA)], and argued that the open-cell design might be associated with fewer radiofrequency shielding artifact^24^. The Alpha stent is basically a closed-cell type, and it may be more susceptible to MR artifacts than the Enterprise stent. Although several studies have discussed the relatively poor ability of TOF-MRA to visualize in-stent flow, its role as a noninvasive tool for evaluating the residual lumen of aneurysms is generally accepted^25–27^. In our study, residual flow to the aneurysm and the patency of adjacent arteries were well visualized by TOF-MRA (Fig. 4). However, in-stent stenosis may not be sufficiently assessable using TOF-MRA alone. Ultrashort echo time-MRA is reportedly a useful noninvasive tool for follow-up of stent-assisted coil embolization for cerebral aneurysms due to its superior visibility over the conventional TOF-MRA^28,29^. Future study should investigate whether the newer MR sequence can improve the suboptimal visualization of the intra-aneurysmal or in-stent flow associated with the Alpha stent™.

This study had several limitations. First, the small study population and short follow-up period limit the generalizability of our results with regard to the long-term safety and efficacy of the Alpha stent. Although 6-month radiological follow-up is relatively short, the interval is commonly chosen in the evaluation of new devices. Second, the size of the included aneurysms was relatively small; the long-term efficacy of the Alpha stent should be further verified in larger wide-necked aneurysms. Third, follow-up radiologic evaluations were conducted using non-contrast TOF-MRA only. As previously mentioned, TOF-MRA is regarded as a standard tool for the follow-up evaluation of aneurysms treated by SAC considering the noninvasive nature of cerebral angiography, although in-stent flow may not be fully assessed. Finally, we included only patients with unruptured ICA aneurysms; comparison of our results to those of other studies that included IAs in various locations should be interpreted with caution, and our results may not be applicable for the treatment of ruptured aneurysms or aneurysms at other locations.

## Conclusion

In this prospective, single-arm study, SAC embolization of unruptured distal ICA aneurysms using the modified Alpha stent has exhibited excellent results regarding safety and efficacy. However, radiologic follow-up by TOF-MRA demonstrated suboptimal visualization of in-stent flow. Further studies on its use in various locations with long-term clinical and radiological data are necessary.

## Data Availability

The datasets used in the current study are available from the corresponding author on reasonable request.
